# Workplace Inclusion of People With Health Issues, Immigrants, and Unemployed Youths—A Qualitative Study of Norwegian Leaders’ Experiences

**DOI:** 10.3389/fpsyg.2022.687384

**Published:** 2022-06-16

**Authors:** Tone Langjordet Johnsen, Tonje Fyhn, Anika Jordbru, Steffen Torp, Torill Helene Tveito, Irene Øyeflaten

**Affiliations:** ^1^Department of Physical Medicine and Rehabilitation, Vestfold Hospital Trust, Tønsberg, Norway; ^2^NORCE Norwegian Research Centre, Bergen, Norway; ^3^Department of Sport, Physical Education and Outdoor Life Studies, University of South-Eastern Norway, Borre, Norway; ^4^Department of Health, Social and Welfare Studies, University of South-Eastern Norway, Borre, Norway; ^5^National Advisory Unit on Occupational Rehabilitation, Rauland, Norway

**Keywords:** workplace inclusion, diversity, leadership, marginalization, leader-member exchange

## Abstract

**Aim:**

To explore leaders’ perceptions and experiences of facilitators and barriers for successful workplace inclusion of immigrants, unemployed youths, and people who are outside the labor market due to health issues.

**Methods:**

Semi-structured individual interviews with 16 leaders who actively engaged in inclusion work, representing different occupations, were conducted. Systematic Text Condensation was used to structure the analysis.

**Results:**

The participating leaders emphasized that job match, including their perception of workers’ motivation, respect for workplace policies, and the availability of appropriate accommodation at the workplace, facilitated work inclusion. An active public support system providing professional and financial support to workers and leaders was also an important facilitating factor. The leaders emphasized that their perception of workers’ lack of motivation for the job was the most important barrier in their own hiring and inclusion engagement. Successful inclusion depended on all workers acknowledging responsibility for and contributing to an inclusive work environment. Being open and willing to discuss challenges was an important part of making the inclusion work. In addition, leadership qualities, such as empathy, patience, and a non-judgmental attitude, appeared as a hallmark among these leaders who actively engaged in inclusion work.

**Conclusion:**

Workplace inclusion of this population of marginalized people was facilitated by job match, mutual respect, commitment, and trust, as well as financial and practical support from the public support system. Leaders’ inclusion practices were furthermore affected by personal attitudes and perceptions of social responsibility. Even so, successful workplace inclusion was presented as a two-way street. Leaders have the main responsibility in initiating a respectful and trusting relationship, but both the worker and the leader needs to contribute to make the relationship thrive.

## Introduction

Preventing labor market exclusion of marginalized groups, that is, people who experience discrimination and exclusion because of unequal power relationships across social, cultural, political, and economic dimensions, is crucial to strengthen equality of opportunities, increase effective labor supply, and reduce public expenditure (OECD, 2010, 2021). Decent work for all, full and productive employment is a part of the United Nations Sustainable Development Goals (United Nations, 2020). Consequently, recruitment and retention of workers with increased likelihood of discrimination has gained growing attention (Ferdman and Deane, 2014). In Norway, the “agreement for a more inclusive working life” (the IA agreement) was first launched by the government in 2001 (The Norwegian Government, 2014). The IA agreement is a tripartite agreement on the national level between the government, the employers’ organizations, and the unions, making workplaces a priority setting for inclusive practices. This includes a focus on integration of marginalized groups. In this study, marginalized groups refer to people who experience exclusion due to mental health issues, impaired functional ability or social challenges, immigrants, or unemployed youths, mainly engaged in work training and vocational rehabilitation services.

From the individual perspective, work is more than an income, and if individuals lose their job, or never get the opportunity to enter the labor market, it may impact their health and wellbeing. Having a job gives people an entrance to the communities they live in and allows them to contribute to those communities. Work provides social contact and opportunities for personal growth, gives identity, status, meaning, and purpose in life (Perkins and Repper, 2013). Employment and socioeconomic status are the main drives of social gradients in health and mortality (Waddell and Burton, 2006; Mackenbach et al., 2008), and several studies have concluded that work is generally good for our health and wellbeing, especially for our mental health (Bennett, 1970; Rowland and Perkins, 1988; Waddell and Burton, 2006; Fossey and Harvey, 2010; Perkins, 2012; van der Noordt et al., 2014). Accordingly, there are both individual, economic, social, and moral arguments to promote employment and focus on workplace inclusion of marginalized groups.

The term inclusion is grounded in how we handle the representation of multiple types of differences at the workplace and is considered a key approach to reap the benefits of diversity (Ferdman and Deane, 2014). Inclusive workplaces involve creating a work environment where workers’ uniqueness and differences are recognized, appreciated, and treated as an advantage for the organization (Roberson, 2006). It is therefore closely linked with organizational culture (Findler et al., 2007). Although organizational culture is shaped by numerous external and internal factors, the impact of leadership on culture is regarded as crucial (Schein, 2010). At the same time, leadership behavior is shaped by the organizational culture (Schein, 2010). Accordingly, workplace inclusion is facilitated both by coworkers and leaders, by the values and norms functioning at the workplace, in addition to the individuals’ own behavior and attitudes (Spataro, 2005; Ferdman and Deane, 2014). Furthermore, a good job match that benefits both the employer and the job seeker is considered an important factor for job satisfaction and job tenure (Becker et al., 1996, 2015; Mueser et al., 2001). Job match is a term used in the vocational rehabilitation literature to capture the key aspect of obtaining long-term work participation for people with some form of disability (Nützi et al., 2017). It is related to terms used in management literature, such as person-organization fit, but is more comprehensive as health-specific needs in addition to personal characteristics, capabilities, and skills are considered in combination with the requirements and needs of the workplace (Carlson et al., 2008).

Several scholars have suggested specific types of diversity “lenses” that explain different ways of approaching diversity in organizations. The evidence base for these conclusions range from small case studies (Ely and Thomas, 2001) and findings from focus groups (Fraser et al., 2010), to more extensive data collections (Podsiadlowski et al., 2013). In a review of diversity studies, Dwertmann et al. (2016) categorize approaches to studying diversity into the fairness perspective and the synergy perspective. While the fairness perspective focuses on equal opportunity and elimination of social exclusion, the synergy perspective focuses on releasing the potential of added value in a diverse workforce (Dwertmann et al., 2016). It is reasonable to assume that a leader emphasizing fairness in recruitment practices may not have great expectations toward the potential benefits of workforce diversity. On the other hand, a leader motivated by the synergy perspective will recruit diversely based on an expectation that it will generate added value for the company, such as a positive work environment and better problem-solving (Dwertmann et al., 2016). Although the two perspectives are clarifying in terms of explaining how leaders deal with diversity in the workplace, they fail to explain how leaders successfully foster a well-functioning work environment within the paradoxes that may arise over time.

A paradoxical perspective focuses on how organizations can attend to competing demands simultaneously (Smith and Lewis, 2011). Adopting a paradoxical perspective and addressing both the benefits and challenges of workplace inclusion is both useful and necessary (Ferdman, 2017). At its core, inclusion involves the coexistence of contradictory facets, handling the fluctuations of performance and attendance in a workforce characterized by functional and health-related diversity. Examples of these contradictory facets are close follow-up versus personal responsibility, managing work demands versus providing flexibility, and functioning versus relapses of illness symptoms (Ferdman, 2017). Creating inclusive workplaces, and benefitting from it, is likely to require the ability to “function with paradoxes in productive ways” (Ferdman, 2017).

Leaders have a particularly important role in fostering inclusion, a role that goes beyond the interpersonal behaviors among coworkers (Shore et al., 2010). This includes making connections between organizational goals and inclusion and holding others responsible for their behavior. In many ways, inclusive leadership is a prerequisite for facilitation of workplace diversity and inclusion (Shore et al., 2010). Thus, it is especially interesting to explore leaders’ experiences and perspectives on successful inclusive practices. When leaders understand and work toward inclusion, they may recognize multiple benefits of diversity and become more concerned with eliminating bias and discrimination (Ferdman and Deane, 2014). These experiences may contribute to the development of valuable skills that have personal and organizational benefits, and if shared, be of value to others as well.

To explore how leaders manage inclusion practices, the theory of leader-member exchange may provide a useful lens. According to the leader-member exchange (LMX) theory (Graen and Uhl-Bien, 1995), leaders develop different levels of exchange with workers, ranging from high-quality to low-quality relationships. High-quality relationships are based on mutual respect, trust, and loyalty, and this type of relationship between leader and worker is found to have positive implications for job satisfaction, organizational commitment, task performance, and organizational citizenship behavior (Dulebohn et al., 2012). Some workers may need and benefit more from high-quality LMX than others (Ferdman, 2017). For workers belonging to the demographic minorities at the workplace, high-quality LMX may foster higher psychological safety, motivation, confidence to speak up and to behave authentically (Gómez and Rosen, 2001; Polzer et al., 2002). The LMX theory point out that it may not be easy for leaders to avoid an in-group/out-group differentiation, due to workers’ individual characteristics. Colella and Varma (2001) showed that leaders generally develop lower quality relationships with disabled workers, indicating that leaders should be especially attentive not to differentiate between workers based on unfair or stereotype-laden grounds. Furthermore, when leaders treat workers as an important member of the organization, coworkers become more willing to accept that person as a valuable member of the group (Brimhall et al., 2014). When workers feel valued and accepted by leaders and coworkers, they feel more included (Shore et al., 2010). Part of the success in fostering workplace inclusion is likely to be determined by the quality of interaction between leaders and workers (Shore et al., 2010; Brimhall et al., 2017). Through this relational dimension of leadership, leaders may influence how workers evaluate themselves in their work environment and their perception of organizational inclusion.

The aim of the current study was to explore how Norwegian leaders experience workplace inclusion of people with health issues, immigrants, or unemployed youths. What is their rationale for making the effort with inclusion and what do they see as facilitators and barriers to creating an inclusive work environment in their organization?

This study took place within the Norwegian work context. Norway has a well-developed public support system facilitating workplace inclusion of marginalized groups. The Norwegian Welfare and Labor Administration (NAV) is a public agency for all Norwegians, administrating a third of the national budget through schemes, such as unemployment benefits, work assessment allowance, sickness benefits, and different employment support arrangements (NAV, 2019). NAV’s main goals are to contribute to a well-functioning job market with a comprehensive and efficient labor and welfare administration, to provide good services tailored to users’ needs, and to help more people to be active and stay at work (NAV, 2019). The main assignment of the NAV Employment Centre is to aid the workplaces to comply with the IA agreement of inclusive workplaces through promotion of good work environments and skills enhancements.

Through the different employment schemes provided by NAV or other private and public vocational rehabilitation services financed by NAV, individuals and employers may receive both financial and practical support. Individuals get the necessary support to find work, help with work adaptation, and the opportunity to try working in ordinary jobs. Individuals who are disabled or unemployed may receive work training or work as trainees to acquire work experience and necessary skills (i.e., entry-level roles). The opportunity to receive these work support programs are the same for everyone, regardless of the reason for being excluded from work. Workplaces who are willing to hire workers who need extra support to enter the labor market may apply for an inclusion subsidy or wage subsidy from NAV for a limited period. The intention of this subsidy scheme is to facilitate recruitment and hiring of job applicants who need vocational follow-up. The traditional effort to increase labor market participation in Norway has been the train-then-place approach (Spjelkavik, 2012; Nøkleby et al., 2017; Sveinsdottir et al., 2019), where individuals are trained in work-related tasks before attempting to enter the competitive labor market (Corrigan, 2001). However, the use of the place-then-train approach (Drake et al., 2012), focusing on direct placement into relevant paid work in a competitive job setting, is increasing.

## Materials and Methods

In this study, data was collected through semi-structured qualitative interviews with Norwegian leaders. By asking leaders about their specific workplace inclusion practices, we wanted to explore their experience with facilitating factors and barriers to successful workplace inclusion of marginalized groups.

### Recruitment and Sample

Sixteen leaders who had relevant experience with employing people who faced challenges in entering the labor market were recruited. To obtain rich and concrete data, the participants were chosen through purposive sampling, seeking leaders who actively engaged in workplace inclusion. Eligible companies in the eastern or western part of Norway were identified through the NAV Employment Support Centre. The NAV Employment Support Centre provided a list of potential candidates, with name and contact information of the leader. These companies were perceived as good candidates because of their previous practices and cooperation with the Employment Support Centre in NAV. When available, public information, such as web sites, was used to gather additional information about the company. The identified workplaces were then contacted by email or phone by three of the authors (AJ, ST, and IØ), and given information about the study and an invitation to participate. The information highlighted that processing of personal data from the interviews (both on the employer and the employee side) would be handled confidentially and that workplaces and the participating leaders would be anonymous. Twenty companies were contacted. One company denied the invitation and three companies did not respond. Sixteen companies approved the invitation and appointed a suitable leader (e.g., human resources manager or general manager with personnel responsibility) to represent the workplace. The sample consisted of eight men and eight women. Fourteen of the leaders worked in the private sector and two in the public sector. Five of the leaders worked in small size companies (<50 employees), eight leaders worked in medium-size companies (50–249 employees), and three leaders worked in large size companies (>250 employees).

### Data Collection

Data were drawn from individual interviews with recruited leaders, lasting between 60 and 90 min. The interviews took place at the participants’ workplaces and were conducted by one of the recruiting authors (AJ, ST, and IØ). To inform which topics to cover as a minimum, a semi-structured interview guide was used (Brinkmann and Kvale, 2015). The interview guide invited participants to share experiences and talk about their workplace inclusion practices, the positive and negative consequences of diversity, facilitators and barriers, and cooperation with the support system. The interviews were audio-recorded, anonymized, and verbatim transcribed.

Sample size was guided by assessment of information power, as described by Malterud et al. (2015). Information power indicates that adequate sample size is judged by the amount of information the sample holds relevant to the study aim and depends on the specificity of the sample and research question, the quality of the dialogue, application of a theoretical framework, and the analysis strategy (Malterud et al., 2015). The current study had a specific research question, and because of their active engagement in workplace inclusion, the sample was highly relevant for the study aim. The dialog quality in the interviews was good, the participants shared plenty of experiences relevant to the study aim, and the study was supported by established theory. After 16 interviews we concluded that the information power was sufficient to conduct a responsible analysis and to answer the specific research question.

### Analysis

The transcripts were analyzed using Systematic Text Condensation (STC), an explorative, thematic, cross-case method for qualitative data analysis, following a stepwise approach (Malterud, 2012). STC has its origin from the descriptive phenomenological method of Giorgi, looking at objects from the perspective of how they are experienced in the search for the essence of the phenomenon (Giorgi, 1985, 2009). STC is a descriptive approach, with the ambition to present vital examples from the informants’ world (Malterud, 2012). The current analysis focused on participants’ experiences with employing individuals with a challenging history or current situation (e.g., history of drug addiction or current mental health problems) and their rationale for making the effort with inclusion. The analysis incorporated the following four steps:

1. *General impression of the material*: all authors participated in the first step reading the transcribed interviews to identify preliminary themes. During this process, the researchers consciously tried to bracket out their preconceptions. The authors met to share and discuss their general impression and agreed on six preliminary themes. The themes were “diversity is a win-win situation,” “job match,” “personal commitment,” “structure and limits,” “support network,” and “diversity challenges.”
2. *Developing code groups based on the preliminary themes*: three of the authors (TJ, AJ, and IØ) met during the second step to identify units of meaning related to the code groups. This was a comprehensive, non-linear process identifying and categorizing meaning units in the transcripts related to the code groups. The researchers reflected and negotiated on what each meaning unit was about and under which code it belonged, and whether the naming of the code groups were suitable. The need to reduce the number of code groups was also an issue in this step.
3. *Condensation of meaning*: the third step in the analysis comprised further development of the results by establishing subgroups and condense the meaning units by rewriting them into a first-person narrative, representing a sum of the participants’ voices described in the data.
4. *Synthesizing*: the last step entailed synthesizing the content of each code group to present a reconceptualized description of each condensate regarding leaders’ experiences with workplace inclusion of marginalized groups. The same three authors (TJ, AJ, and IØ) met and collaborated, performing the two last steps of the analyses before the rest of the research team were invited to comment in several rounds. Agreement on four final core themes was reached (see “Results”). The LMX theory (Graen and Uhl-Bien, 1995) was used as a theoretical framework to sharpen the focus for interpretation of results and discussions. The analysis was not theory-driven with prearranged coding, but inductive and iterative.

### Ethics

The study adhered to the Declaration of Helsinki (WMA, 2013) and was approved by the Norwegian Social Science Data Services (NSD, ID 53262). All participants gave their informed consent to participate in the study.

## Results

The structured analysis process established various relevant perspectives on how leaders perceived and facilitated workplace inclusion of people who faced challenges in entering the labor market. Three main groups emerged from their practice experience; people with different health issues, immigrants, and unemployed youths. The participants stressed that they considered diversity primarily as a benefit for the workplace. Giving individuals the opportunity to work and feel included in a work environment, resulted in individual as well as organizational gains. Another motivation for facilitating inclusion was corporate social responsibility. Showing empathy, patience, and having a non-judgmental attitude were highlighted as important leadership qualities when hiring individuals in need of extra follow-up and support. Furthermore, participants emphasized that job match, exemplified through individual motivation, respect for workplace policies, and availability of appropriate accommodation, and an active public support system were important facilitating factors for successful inclusion.

Through the analysis, four core themes were found. These themes are elaborated below, by synthesizing the relevant statements from each interview into a coherent summary. As described in the analysis section, the summary closely resembles the raw data.

### Diversity Practice Is a Win-Win Endeavor

The participants clearly considered diversity a benefit for the workplace as well as for the individual. They described it as a win-win situation where the workplace got motivated workers, and individuals flourished as they were assigned to new tasks and responsibilities. One of the leaders emphasized that the more a person thrives at work, the more that person contributes to the workplace. The leaders found it rewarding to watch how being part of a work environment made many of their new workers gain confidence, as they went from receiving welfare benefits to paying taxes. Several of the leaders also considered inclusion efforts to be an investment in reducing sick leave at the workplace. They reasoned that including people who had health limitations, but had positive work attitudes and job engagement, motivated other workers and increased the threshold for taking sick leave. One leader gave an example from his workplace. He had hired a worker with a chronic condition and it took the worker a lot of effort and energy just to get from home to the workplace. He also needed a break in the middle of the day, but always returned to work. The leader said this dedicated coworker, who always did his best, was a positive influence on other workers. Some of the participants described similar benefits from ethnic diversity. One leader mentioned that workers from other countries were highly reliable, had few days of sick leave and high work ethics. Such work attitudes influenced other workers, which again made the leaders more positive to recruit immigrant workers. One participant summarized the benefits of workplace inclusion for his company like this:

*“I believe the customers approve and accordingly it improves the reputation of my business and thus is an investment for me. (…) My personal reward is to contribute to a better society, to make my day easier, and last, but not least, my product gets better.”*

The individual benefits of employment were evident in many of the participants’ stories, where they perceived employment to be a turning point, especially for their young workers. They were described as a vulnerable group, often with a difficult life situation. Many had issues related to mental health and social functioning that had hindered their entry into the labor force. Initially, these workers needed close follow-up and non-demanding tasks, until they found their place in the company. Although this could take a long time, the process often ended with a competent and loyal worker with high work capacity. One leader told a story of a young man with mental health issues who had lived with his parents and spent all his time playing video games. When he started working, he did not look people in the eyes, the leader said. But gradually he became more self-confident, until he expressed almost on a daily basis how grateful he was for his job. Another leader told about a young person who had been in prison for committing drug-related crimes. This person showed a lot of initiative at work and the leader provided relevant literature and online courses for him. He studied with great enthusiasm, the leader said, and for the last 4 years, he had been employed on ordinary terms. A third leader described how a young worker changed her name after a process of personal development, as a way to leave her old self:

*“It was amazing to see an individual evolve from a nervous girl, working for two hours two days a week, to working one-hundred-percent and shine like the sun.”*

Many of these workers entered work life with poor prospects, but after one or 2 years they had gained work experience, a work certificate, a certificate of apprenticeship, proof of competence, an improved CV, or a contract of employment. According to the leaders, the keys to success were openness and willingness from the worker to share their difficulties so they could discuss different solutions together. In this way, individuals got a chance to work out their issues while participating in working life. One leader summarized the two-fold benefits of diversity and inclusion in these words:

*“There are tremendous resources out there, and they are not utilized. Giving individuals an opportunity to return to work with all its benefits – that can make a huge difference in a human life.”*

### Employment—a Mutual Dependence

Several participants expressed that they perceived their workplace to be inclusive, but said that for successful inclusion to occur, the job seekers themselves also had to want the job. A recurring topic in the interviews was that the leaders wanted workers to show willingness and motivation, to some regard needed them to carry out their work tasks, and generally show respect for the workplace conditions. These topics were rooted in both previous experiences and their commitment to running a company. Some of the participants had experienced that some workers were sent around by vocational rehabilitation services from workplace to workplace without success, but these leaders perceived the real barrier to be that the workers did not want to work. Based on these experiences, they always explored any job seeker’s motivation in advance. If behaviors and attitudes of the job seeker were interpreted as a lack of motivation, this was a barrier for employing the person; while age, ethnic background, and physical or mental health challenges were not considered barriers. The participants gave many examples of situations where motivated workers had contributed to successful work inclusion. One leader told a story about a refugee who started an internship in their company. He showed great interest in one of the machines, and because of this interest, he was almost fully trained to operate the machine after six months. When the company had a vacancy, he applied and was hired. Another leader told about a worker with a serious health condition and explained that if he had not wanted to work, he would not be part of the labor force, because nobody would expect that from a person with his limitations. He stated:

*“I can’t sit here and claim that we have included him because we are so good. He wants to be here, and then we can facilitate to his needs.”*

When hiring, the leaders were looking for the applicant’s competence to do the job. They explained that they depended on all workers doing their share of the work tasks and needed to trust that the customers would receive a satisfactory product. Several of the leaders expressed that unsuccessful inclusion often was due to a poor job match. A leader gave an example of a worker she had hired to work in the reception, where mastery of her new language had been a barrier. The worker talked, she said, but no one understood what she was trying to say. For a reception job, where the most important task was to provide good service to customers, this was not a good match at that time. Similarly, a leader in a craft business said that it would be difficult for them to include persons with physical handicaps, like for instance a blind person. Generally, the leaders were over-bearing, but also set limits, as stated by one of the participants:

*“We need to express clear expectations. We cannot have wide open doors. We do this because we think the person in question may fit the job.”*

Some of the leaders also mentioned that they needed workers to adhere to the conditions at the workplace, meaning that they had to relate to the companies’ opening hours and to supervisors of a different gender or cultural background. If there was a poor match between such workplace conditions and the employees’ wishes, the leaders saw it as best for both parts not to employ them. When it came to culturally diverse workplaces, several leaders mentioned that there were some specific issues to be aware of. Common examples of such issues were unwillingness among men to accept supervision from a woman, or unwillingness to accept supervision by someone from another cultural background. This was not acceptable to the leaders. They saw it as important that coworkers accepted and treated each other with respect at work, despite different gender, ethnicity, or religious beliefs. Similarly, they wanted workers to respect the Norwegian work culture, where for instance equality is an important value. One leader told about a worker who brought her husband to work, where the husband spoke on her behalf. He stated:

*“We were annoyed about that, because he had no business to be here (…). Workers must be allowed to speak for themselves. We told her that we wanted her here, but that she had to respect our rules.”*

Another challenge mentioned by the participants was that people with the same background tended to form subgroups. Some of the participants explained that because of this tendency they deliberately hired workers from many different cultures. The main issue was that subgroup members would protect each other by not talking to their leader if something happened at work, and this made integration difficult. To avoid uncomfortable and difficult situations, several leaders explained that they had implemented a rule where all workers had to speak Norwegian around other coworkers. This was a measure done to integrate all workers and to make sure everyone could understand what was being said. Another company made similar choices regarding job seekers with a history of drug addiction. The leader explained:

*“We cannot create a workplace with many former drug addicts in respect for those who have succeeded. The biggest challenge for many drug addicts is that they do not want contact with their former environment and to succeed with the one we have included already, we must close the door for others.”*

### Successful Work Inclusion Involves Committed and Empathic Leaders

The leaders were committed to taking social responsibility and determined to be patient and understanding toward workers in need of additional support. Several of the leaders explained that very much in their company was governed by their own feelings and opinions, and they shared how their own life experience had led to their commitment to creating inclusive workplaces. One of the leaders called himself a drop-out kid and “a lonely wolf,” thus he created a business with a culture where youths with similar challenges were welcome to work. Efforts to increase employment opportunities for a broader range of people were perceived as taking social responsibility. Most of the people who were accepted for work training *via* vocational rehabilitation services had impaired functional ability or social challenges and required quite a lot of follow-up and instruction. The costs associated with having someone in training did not match the compensation that workplaces received from the welfare system, thus the incentives were not financial. The incentives were to help, as expressed by one of the leaders:

*“I have an intention to give something back to society. This is very important to me, and I am not just saying that – this is something I feel we should do.”*

Successful inclusion practices were associated with various leadership traits and behaviors. Many participants emphasized that an important part of a leader’s job was to provide growth opportunities, security, encourage open dialogue, have a positive, non-judgmental attitude, and not expect new workers to manage everything the first week. They saw it as their own responsibility to create a culture where coworkers take care of each other, and where workers are confident enough to try and thereby risk making mistakes. Another important characteristic was to be patient and not give up on someone, even when the easiest solution was to let them go. One of the participants told a story about a worker who had very low self-esteem and frequent absenteeism, but instead of giving up on him, they gave him responsibilities and sent him on a course abroad. This worker had never been abroad and never received opportunities like that. The leader explained that this experience had changed the life of this worker, because he had been trusted, felt that he was good enough, and experienced a feeling of mastery. Other participants also expressed that they would rather push someone to stay and make it work than let them go, because they did not want to destroy people who already were in a difficult situation. They believed that work could contribute to meeting important human needs, like achieving personal goals, responsibility, and trust, and generally assumed that everybody wanted to work if they got the chance. Furthermore, leaders expressed willingness to accommodate and adjust work tasks if the workers were open about their specific challenges. One leader said that he would not mind giving a worker with former alcohol problems a day off if he fell off the wagon on the weekend, but it would be unacceptable for him to show up to work intoxicated. Willingness to give people a chance, despite their health problems or background, was frequently mentioned. One leader summarized it in these words:

*“I think it’s important to let people try. And that is completely independent of where they come from or where they are going. I think we should treat everybody equally. It’s nice to be able to give people an opportunity.”*

Participants emphasized the importance of building an organizational culture where it was natural to work with inclusion in a systematic way, and identifying those leaders who saw inclusion as a value and focused on resources rather than limitations. One of the participants explained that if they had twenty leaders in the company and ten of them perceived inclusion as a good idea, they did not worry about the remaining ten. They built a culture around the ten leaders who thought inclusion was important, and the message naturally spread when leaders and workers talked and cooperated. Several of the leaders had experienced that coworkers who got involved were very interested in providing help and support if they got the chance to do so. A recurring topic among the participants was that workplaces who were in a position to offer someone a job or training, a work environment, and a place where someone was waiting for them in the morning, should strive to do so. It was possible to have a capitalistic point of view, with interpersonal values. One participant stated that it should be a corporate responsibility for companies over a certain size to include those outside the labor market. He justified this statement as follows:

*“Because it quickly becomes a vicious circle; they do not get the chance to enter the labor market because they lack work training, and they do not get an ordinary job without work experience.”*

### Workplaces Need Assistance From the Public Support System

The need for support when working with inclusion of workers with challenges was repeatedly mentioned by the participants. They emphasized that in order to create an inclusive work life, it was not enough that the workplaces provided time and resources. They also needed systematic follow-up and active support from NAV or the vocational rehabilitation services NAV collaborates with. Many of the participants described that they cooperated with NAV or other vocational rehabilitation services, and worked systematically with the inclusion of candidates for work training and employment. They further explained that in the period a worker had work training and a position as a trainee, he or she received benefits from NAV in lieu of a salary from the company. This enabled the workplaces to give trainees enough time to learn tasks and to provide extra support and adjustments when needed. The leaders stressed the importance of onboarding new workers properly, integrating them into a work group, and not leave them to themselves. One leader told about a worker on tryout for permanent part-time employment in the company. This worker had a certain medical condition requiring a great deal of predictability and was in need of close follow-up. Every day, the leader would thoroughly describe today’s tasks, provide him with a list of things to do, and at the end of the day, they would talk through tasks for the next day. Because of the extra effort required in situations like this, several participants expressed that financial and professional support was crucial to help them carry the extra load. Without this support, the risk would be too large for most companies. One of the participants clearly expressed what he could contribute with, and what the system should contribute with:

*“My contribution is my time, my employees’ time and an office we can use. I contribute with care and help people get a job. Then it is important that those who work at the vocational rehabilitation services or the refugee center facilitate to make it easier for employers.”*

Generally, the participants expressed satisfaction with the support system and experienced it as a positive incentive for leaders to increase diversity in the workplace. However, one issue was mentioned by several of the participants. Most of the leaders wanted to recruit in a long-term perspective, and wished for a more long-term support perspective, adjusted to the needs of the workers and workplaces. One of the leaders explained that when the company had a trainee working for a longer period of time, he or she had the possibility to really learn the work tasks, and for example try out being alone in the reception while coworkers took their lunch break. If the subsidized practice period was extended, several leaders meant that it would become more of a win-win situation. Furthermore, leaders stressed that when they had trainees who performed well, they did not want to dismiss them just because they were not able to contribute a 100%. One of the participants gave an example of a worker who had worked there for 2 years, partly with support from NAV. This worker had struggled with anxiety and depression for over 20 years. Even though he now was functioning better, his health complaints did not disappear, and the work situation was still challenging. Some of the participants stated that if companies could continue to receive a small compensation from the system in situations like this, it would be easier to give workers with limited function permanent jobs. In general, a trainee position was seen as a great opportunity for transition to ordinary work. Because of the good cooperation between the vocational rehabilitation services and the companies, many of the leaders said that they rarely advertised available positions. Thus, marginalized workers got the opportunity to skip the two most difficult steps in the hiring process, in terms of submitting an application and doing a traditional interview. One of the participants gave a description of their current experience with the support system:

*“When this cooperation has lasted for a while, they (the vocational rehabilitation services) start to know us very well – they are good at choosing the right trainees for us. This has been a great strength. We have received help to take care of trainees during the training phase, and assistance to handle the difficult things.”*

## Discussion

Our analysis demonstrated that the leaders in this study, who were invited to participate due to their active engagement in inclusion work, considered workplace inclusion as a benefit for the organization as well as for the individual. The analysis further indicated that certain leadership qualities, such as understanding, trust, and respect, created a foundation for successful inclusion efforts, but also that workplace qualities, such as willingness and ability to accommodate worker needs, were important ingredients for sustainable inclusion efforts. However, the leaders did emphasize that workplace inclusion did not depend only on the ability and effort from the leader and the workplace to accommodate worker needs. They pointed out that the workers also had to engage in the establishment of a good work relationship and highlighted the workers’ motivation, their respect for norms and rules, and openness about their challenges and limitations as valuable contributors in this regard. Lastly, it was emphasized that good cooperation between the workplaces and the public support system was crucial.

### Relational Dimensions of Leadership in Inclusion Practices

In line with the LMX theory, the relational dimension of leadership was prominent in the results. This study indicates that traits of the leader, such as understanding, determination, and commitment, may be valuable components in the effort to succeed with workplace inclusion of persons with mental health issues, former drug abuse, impaired functional ability, immigrants, or unemployed youths. Even though each person may encounter a unique set of challenges in their job participation, for example, language barriers may be common among immigrants but not an issue for native speakers who are marginalized for other reasons, the mentioned leader traits seemed to be of high value across the different groups in this study. These traits were illustrated through different actions, such as “tough love,” not giving up on workers who needed time to adjust to working life, acknowledging each worker’s unique competence, and being committed to creating a culture where coworkers take care of each other. The findings in this study coincide with the empirical evidence found in the study of Brimhall et al. (2017), indicating that high-quality leadership interactions are an important component in facilitating workplace inclusion. Along the same line, Shore et al. propose in their model for future research on workplace inclusion that a joint consideration of employee belongingness and uniqueness may advance inclusion practices (Shore et al., 2010). When the workplace and the leader focus on and appreciate the unique characteristics of each worker and the workers are acknowledged as an important part of the organization (belongingness), it may contribute to perceptions of inclusion and improved satisfaction and commitment (Shore et al., 2010). Ensuring that the workers’ competence matches the work tasks, and developing a culture where coworkers support and take care of each other, may be important leadership efforts in creating a feeling of belongingness in the workplace. In this study, leaders believed that their inclusive actions and convictions of social responsibility affected their workers and positively influenced their willingness to include persons having different health or social challenges.

The LMX theory emphasizes that the creation of high-quality LMX is a collaboration. Even though the leader may be the one initiating the exchange, LMX is a two-way street. It requires that both the leader and the worker contribute to the relationship. In the current study, the workers’ contribution to this relationship emerged as an important issue. The leaders found it challenging to hire a person who they perceived as unmotivated to work there or a person who did not respect the workplace conditions (e.g., having a female leader). An important point of discussion is that we do not know what type of organizational culture these leaders are creating; is it the job seeker that is unmotivated or is it the workplace that lacks the understanding and competence to include people with a history of discrimination and exclusion? In the interviews, the leaders frequently used the term motivated (or unmotivated) about employees. Motivation is a major concept that is difficult to precisely define. It may be interpreted as an attitude, a behavior, or both; as something drawn by internal forces, external forces, or both; and may not necessarily be a good indicator of employees’ job performance (Rainey, 2000). Motivation is a commonly used word in everyday speech, but because of the mentioned complexities, a straightforward interpretation of the meaning may be difficult. In this study, we have no data to help us interpret what the leaders mean when using the term motivated or unmotivated about employees. It seems reasonable to ask if these marginalized workers, with a long history of being outside working life, along the way may have lost self-confidence and belief in their own abilities. Lack of self-confidence and negative expectancies may be perceived as signs of not being motivated. We know from school drop-outs that low self-esteem and low expectations often are related to low intrinsic motivation (Alivernini and Lucidi, 2011). On the other hand, an intolerant attitude among some leaders toward some of their workers may illustrate lack of knowledge about being outside the labor market and how marginalization can contribute to withdrawal (Burke et al., 2013; Lindsay et al., 2014). Therefore, one role for the NAV Employment Centre may be to raise the level of knowledge among leaders on these issues.

### An Active Public Support System

Support from the public support system (NAV) was another important condition mentioned by many of the leaders. This support mainly involved professional support (e.g., to deal with health issues) and financial support (e.g., salary reimbursement). Most of the interviewed leaders represented small or middle-sized companies and explicitly stated that the weight would be too large to carry alone. The participants expressed both positive and negative experiences with this system, in addition to presenting suggestions for improvements. The aim of the present study was not to evaluate the Norwegian public support system, but rather to highlight what leaders experience as success factors in facilitating inclusive workplace practices. However, one of their main concerns was that the time limits of the support was too short, making it difficult for both the leader and the worker to achieve beneficial results. According to LMX research, time is central in forming meaningful relationships (Naidoo et al., 2011) and may be especially important for persons who have been out of the workforce for a long time. To complement the leaders’ efforts and produce long-term beneficial results, some system-level adjustments may be required. NAV is increasingly using the work rehabilitation program Individual Placement and Support (IPS), which provides long-term support for employers and employees, and aims to find suitable job matches in order to obtain sustainable employment in the competitive labor market (Becker and Drake, 2003). This development in the public support system has the potential to mitigate some of the concerns voiced by the leaders in the current study.

### Working With Paradoxes

Our results highlight an important point that has previously been overlooked in the diversity literature; that leaders concerned with inclusion efforts are not only working within the traditionally perceived perspectives of synergy vs. fairness (or other related perspectives). While the synergy perspective explains the incentives for obtaining organizational diversity through the potential for added value that different perspectives can produce, the fairness perspective emphasizes values related to equal opportunities and rights (Dwertmann et al., 2016). Our study suggests that leaders work simultaneously within both perspectives, and strive to balance what appears to be somewhat paradoxical concerns. They have a commitment to social responsibility on the one hand, while they also have a commitment to profit and the company’s bottom line. They have positive expectations to the synergistic effects of diversity, while at the same time acknowledging the effort it takes to achieve this synergy. They firmly believe in the potential of the individual, but also acknowledge that some individuals will not be able to live up to the expectations of a fully productive worker with no impairment of function. Several of the leaders addressed these tensions with a “both/and” rather than an “either/or” approach, thereby managing the coexistence of seemingly contradictory facets. According to Ferdman, (1995), balancing the paradoxes is generally more productive than becoming polarized, because the nature and dynamics of diversity mean that both perspectives must be addressed to foster inclusion.

### What Does This Study Add?

There is growing evidence supporting the benefits of inclusion, but there is a need for more research on the antecedents to creating inclusive environments (Dwertmann and Boehm, 2015), as well as on leadership and inclusion (Brimhall et al., 2017). As the benefits of inclusion become clear, it is important that leaders understand what facilitates an inclusive work environment. Our study contributes with insight into how and why leaders engage in inclusion practices, mainly by hiring people with diverse and sometimes challenging backgrounds, and what they perceive as facilitating factors. Furthermore, the leaders in this study seem to battle somewhat contradictory challenges, balancing a genuine desire to make a difference for marginalized people and making profits for the company. Such paradoxical concerns, and how to best manage them, is an important issue for future research. Given inherent market forces, a transforming work life, and an increased awareness of the importance of ordinary work employment, the management of these paradoxes are likely to be an important factor in future successful inclusion practices. Lastly, our study contributes with insight into workplace inclusion practices in the Norwegian context and the importance of the public support system (NAV) in fostering these inclusion practices. However, a main concern raised by the leaders regarding this system was the time constraints that often lie in this support and how the system rigidity may be a barrier for sustainable workplace inclusion. This finding underpins the value of the increased focus we now see on using the work rehabilitation program Individual Placement and Support (IPS), providing long-term support for employers and employees, in addition to focusing on suitable job matches in the competitive labor market in order to obtain sustainable employment.

### Methodological Considerations

Semi-structured individual interviews were chosen since this design is appropriate for collecting data to explore individual experiences from life events and social phenomena, and provides the opportunity for close follow-up of shared experiences (Malterud, 2001). Our sample was balanced regarding gender, consisting of eight men and eight women, who represented different occupations. However, only two of the participants worked in the public sector. Including a greater number of leaders from the public sector would have enriched the data, and given us the opportunity to look for differences in workplace inclusion practices between sectors.

Most of the participants openly presented their experiences, giving the impression of trustworthy and authentic stories. The analysis convinced us that the participating leaders did not glorify their own actions. The answers presenting challenging or negative experiences, and actions calling for improvements, indicated that participants felt safe to report negative experiences as well. Although collaboration and interaction between leaders and workers are important aspects in workplace inclusion, workers were not interviewed. A one-sided perspective in understanding collaboration between multiple actors is a limitation, but since leaders have a particularly important role in fostering inclusion, this study aimed to focus on their experiences. Even so, it is important to bear in mind that what these leaders perceive, and refer to, as inclusive workplaces, not necessarily coincide with what the workers or job seekers need to feel included.

Recommendations for sample size in qualitative studies diverge, but most authors emphasize that there is no magic number. The important issue is whether the sample contains a sufficient amount and variety of events to adequately answer the research question (Malterud et al., 2015). In this study, sample size was guided by information power (Malterud et al., 2015). We concluded that a responsible analysis, drawing on a substantial presentation of relevant events, could be undertaken from the available empirical data. The interview material on leaders’ experiences was rich and diverse, and we consider our findings to be a valuable contribution to the research literature on workplace inclusion and the role of the leader in these processes. The Norwegian welfare system is in some ways unique, but have similarities with the systems in several other countries, such as other Nordic countries and the Netherlands, which strengthens the external validity of our findings.

## Conclusion

Our findings indicate that a good job match, including the worker being motivated for the job, respect for workplace policies, and availability of appropriate accommodation, may facilitate workplace inclusion of persons with mental health issues, former drug abuse, impaired functional ability, immigrants, or unemployed youth. The leaders emphasized that their perception of workers’ lack of motivation for the job was a barrier in their own hiring and inclusion engagement, while mutual trust, commitment, and respect between worker and leader were perceived as important facilitators. Furthermore, they pointed out that patience and a non-judgmental attitude were valuable leader traits when hiring persons with work or social challenges. An active public support system, providing both professional and financial support, contributed to this effort. Leaders’ engagement in inclusion practices were affected by personal attitudes and their understanding of social responsibility, and the leaders perceived that their attitudes and behavior influenced the coworkers’ behavior. Workplace inclusion may generate some tension in the organization, but by embracing the coexistence of contradictory facets and opposing views, one may be better equipped to reap the benefits of diversity dynamics at the workplace.

## Data Availability Statement

The dataset presented in this article is not readily available because of ethical reasons. The study consent ensured participants that the raw data would not be shared beyond the research team to protect their confidentiality. Questions regarding the datasets should be directed to Tone Langjordet Johnsen, email: tojo@norceresearch.no.

## Ethics Statement

The studies involving human participants were reviewed and approved by Norwegian Social Science Data Services (NSD, ID 53262). The patients/participants provided their written informed consent to participate in this study.

## Author Contributions

AJ, ST, and IØ performed the interviews. TJ, AJ, and IØ conducted the analysis. TJ, TF, AJ, ST, TT, and IØ were involved in preparing and revising the manuscript. All authors contributed to the article and approved the submitted version.

## Funding

The study was funded through a research grant from the Research Council of Norway (grant number 255046).

## Conflict of Interest

The authors declare that the research was conducted in the absence of any commercial or financial relationships that could be construed as a potential conflict of interest.

## Publisher’s Note

All claims expressed in this article are solely those of the authors and do not necessarily represent those of their affiliated organizations, or those of the publisher, the editors and the reviewers. Any product that may be evaluated in this article, or claim that may be made by its manufacturer, is not guaranteed or endorsed by the publisher.

## References

[ref1] Alivernini F. Lucidi F. (2011). Relationship between social context, self-efficacy, motivation, academic achievement, and intention to drop out of high school: a longitudinal study. J. Educ. Res. 104, 241–252. doi: 10.1080/00220671003728062

[ref2] Becker D. R. Drake R. E. (2003). A Working life for People with Severe mental Illness. London: Oxford University Press.

[ref3] Becker D. R. Drake R. E. Farabaugh A. Bond G. R. (1996). Job preferences of clients with severe psychiatric disorders participating in supported employment programs. Psychiatr. Serv. 47, 1223–1226. doi: 10.1176/ps.47.11.1223, PMID: 8916240

[ref4] Becker D. R. Swanson S. Reese S. L. Bond G. R. McLeman B. M. (2015). Supported Employment Fidelity Review Manual. 3rd Edn. Lebanon, NH: Dartmouth Psychiatric Research Center.

[ref5] Bennett D. (1970). The value of work in psychiatric rehabilitation. Soc. Psychiatry 5, 224–230. doi: 10.1007/BF00589468

[ref6] Brimhall K. C. Lizano E. L. Mor Barak M. E. (2014). The mediating role of inclusion: A longitudinal study of the effects of leader-member exchange and diversity climate on job satisfaction and intention to leave among child welfare workers. Child Youth Serv. Rev. 40, 79–88. doi: 10.1016/j.childyouth.2014.03.003

[ref7] Brimhall K. C. Mor Barak M. E. Hurlburt M. McArdle J. J. Palinkas L. Henwood B. (2017). Increasing workplace inclusion: The promise of leader-member exchange. Hum. Serv. Organ. Manag. Leadersh. Gov. 41, 222–239. doi: 10.1080/23303131.2016.1251522

[ref8] Brinkmann S. Kvale S. (2015). Interviews: Learning the craft of Qualitative Research Interviewing. Thousand Oaks, CA: SAGE.

[ref9] Burke J. Bezyak J. Fraser R. T. Pete J. Ditchman N. Chan F. (2013). Employers’ attitudes towards hiring and retaining people with disabilities: A review of the literature. Aust. J. Rehabil. Couns. 19, 21–38. doi: 10.1017/jrc.2013.2

[ref10] Carlson L. Smith G. Rapp C. A. (2008). Evaluation of conceptual selling as a job development planning process. Psychiatr. Rehabil. J. 31, 219–225. doi: 10.2975/31.3.2008.219.225, PMID: 18194949

[ref11] Colella A. Varma A. (2001). The impact of subordinate disability on leader-member exchange relationships. Acad. Manag. J. 44, 304–315.

[ref12] Corrigan P. W. (2001). Place-then-train: An alternative service paradigm for persons with psychiatric disabilities. Clin. Psychol. 8, 334–349. doi: 10.1093/clipsy.8.3.334

[ref13] Drake R. E. Bond G. R. Becker D. R. (2012). Individual Placment and Support: An Evidence-based Approach to Supported Employment. New York: Oxford University Press.

[ref14] Dulebohn J. H. Bommer W. H. Liden R. C. Brouer R. L. Ferris G. R. (2012). A meta-analysis of antecedents and consequences of leader-member exchange: integrating the past with an eye toward the future. Aust. J. Manag. 38, 1715–1759. doi: 10.1177/0149206311415280

[ref15] Dwertmann D. J. G. Boehm S. A. (2015). Status matters: The asymmetric effects of supervisor–subordinate disability incongruence and climate for inclusion. Acad. Manag. J. 59, 44–64. doi: 10.5465/amj.2014.0093

[ref16] Dwertmann D. J. G. Nishii L. H. van Knippenberg D. (2016). Disentangling the fairness & discrimination and synergy perspectives on diversity climate: Moving the field forward. Aust. J. Manag. 42, 1136–1168. doi: 10.1177/0149206316630380

[ref17] Ely R. J. Thomas D. A. (2001). Cultural diversity at work: The effects of diversity perspectives on work group processes and outcomes. Adm. Sci. Q. 46, 229–273. doi: 10.2307/2667087

[ref18] Ferdman B. M. (1995). “Cultural identity and diversity in organizations: Bridging the gap between group differences and individual uniqueness,” in Diversity in Organizations: New Perspectives for a Changing Workplace. eds. Chemers M. M. Oskamp S. Costanzo M. A. (United States: Sage Publications, Inc), 37–61.

[ref19] Ferdman B. M. (2017). Paradoxes of inclusion: Understanding and managing the tensions of diversity and multiculturalism. J. Appl. Behav. 53, 235–263. doi: 10.1177/0021886317702608

[ref20] Ferdman B. M. Deane B. R. (2014). Diversity at work: The Practice of Inclusion. San Francisco: John Wiley & Sons, Inc.

[ref21] Findler L. Wind L. H. Barak M. E. M. (2007). The challenge of workforce management in a global society. Adm. Soc. Work. 31, 63–94. doi: 10.1300/J147v31n03_05

[ref22] Fossey E. M. Harvey C. A. (2010). Finding and sustaining employment: A qualitative meta-synthesis of mental health consumer views. Can. J. Occup. Ther. 77, 303–314. doi: 10.1177/000841741007700501, PMID: 21268512

[ref23] Fraser R. T. Johnson K. Hebert J. Ajzen I. Copeland J. Brown P.. (2010). Understanding employers’ hiring intentions in relation to qualified workers with disabilities: preliminary findings. J. Occup. Rehabil. 20, 420–426. doi: 10.1007/s10926-009-9220-1, PMID: 19936892

[ref24] Giorgi A. (1985). “Sketch of a psychological phenomenological method,” in Phenomenology and Psychological Research (Pittsburg, PA: Duquesne University Press), 8–22.

[ref25] Giorgi A. (2009). The Descriptive Phenomenological Method in Psychology: A Modified Husserlian Approach. Pittsburgh, PA: Duquesne University Press.

[ref26] Gómez C. Rosen B. (2001). The leader-member exchange as a link between managerial trust and employee empowerment. G. Organ. Manag. 26, 53–69. doi: 10.1177/1059601101261004

[ref27] Graen G. B. Uhl-Bien M. (1995). Relationship-based approach to leadership: development of leader-member exchange (LMX) theory of leadership over 25 years: applying a multi-level multi-domain perspective. Leadersh. Q. 6, 219–247. doi: 10.1016/1048-9843(95)90036-5

[ref28] Lindsay S. McDougall C. Sanford R. (2014). Exploring supervisors’ attitudes of working with youth engaged in an inclusive employment training program. J. Hum. Dev. Disabil. Soc. Change 20, 12–20.

[ref29] Mackenbach J. P. Stirbu I. Roskam A.-J. R. Schaap M. M. Menvielle G. Leinsalu M.. (2008). Socioeconomic inequalities in health in 22 European countries. N. Engl. J. Med. 358, 2468–2481. doi: 10.1056/NEJMsa0707519, PMID: 18525043

[ref30] Malterud K. (2001). Qualitative research: standards, challenges, and guidelines. Lancet 358, 483–488. doi: 10.1016/S0140-6736(01)05627-6 11513933

[ref31] Malterud K. (2012). Systematic text condensation: A strategy for qualitative analysis. Scand. J. Public Health 40, 795–805. doi: 10.1177/1403494812465030, PMID: 23221918

[ref32] Malterud K. Siersma V. D. Gaussora A. D. (2015). Sample size in qualitative interview studies: guided by information power. Qual. Health Res. 26, 1753–1760. doi: 10.1177/1049732315617444, PMID: 26613970

[ref33] Mueser T. Becker D. R. Kim R. W. (2001). Supported employment, job preferences, job tenure and satisfaction. J. Ment. Health 10, 411–417. doi: 10.1080/09638230120041173

[ref34] Naidoo L. J. Scherbaum C. A. Goldstein H. W. Graen G. B. (2011). A longitudinal examination of the effects of LMX, ability, and differentiation on team performance. J. Bus. Psychol. 26, 347–357. doi: 10.1007/s10869-010-9193-2

[ref35] NAV (2019). What is NAV?: The Norwegian Welfare and Labor Administration. Available at: https://www.nav.no/en/home/about-nav/what-is-nav (Accessed November 5, 2020).

[ref36] Nøkleby H. Blaasvær N. Berg R. (2017). Supported Employment for People with Disabilities: A systematic Review. Oslo: Folkehelseinstituttet. 29553670

[ref37] Nützi M. Trezzini B. Medici L. Schwegler U. (2017). Job matching: An interdisciplinary scoping study with implications for vocational rehabilitation counseling. Rehabil. Psychol. 62, 45–68. doi: 10.1037/rep0000119, PMID: 28206808

[ref38] OECD (2010). Sickness, Disability and work: Breaking the Barriers. A Synthesis of Findings across OECD Countries. Paris: OECD Publishing.

[ref39] OECD (2021). OECD economic Surveys: Norway 2018. Paris: OECD Publishing.

[ref40] Perkins R. (2012). “The right to contribute: employment and recovery,” in From Communism to Schizophrenia and Beyond. eds. McManus G. Carson J. (London: Whiting & Birch), 181–204.

[ref41] Perkins R. Repper J. (2013). Prejudice, discrimination and social exclusion: Reducing the barriers to recovery for people diagnosed with mental health problems in the UK. Neuropsychiatry 3, 377–384. doi: 10.2217/npy.13.34

[ref42] Podsiadlowski A. Gröschke D. Kogler M. Springer C. van der Zee K. (2013). Managing a culturally diverse workforce: diversity perspectives in organizations. Int. J. Intercult. 37, 159–175. doi: 10.1016/j.ijintrel.2012.09.001

[ref43] Polzer J. T. Milton L. P. Swarm W. B. (2002). Capitalizing on diversity: Interpersonal congruence in small work groups. Adm. Sci. Q. 47, 296–324. doi: 10.2307/3094807

[ref44] Rainey H. G. (2000). “Work motivation,” in Handbook of Organizational Behavior, Revised and Expanded. ed. Golembiewski R. T.. 2nd Edn. (New York: Routledge).

[ref45] Roberson Q. M. (2006). Disentangling the meanings of diversity and inclusion in organizations. Group Org. Manag. 31, 212–236. doi: 10.1177/1059601104273064

[ref46] Rowland L. A. Perkins R. E. (1988). You can’t eat, drink and make love eight hours a day: The value of work in psychiatry. Health Trends 20, 75–79.

[ref47] Schein E. H. (2010). Organizational Culture and Leadership. New Jersey: John Wiley & Sons.

[ref48] Shore L. M. Randel A. E. Chung B. G. Dean M. A. Holcombe Ehrhart K. Singh G. (2010). Inclusion and diversity in work groups: A review and model for future research. Aust. J. Manag. 37, 1262–1289. doi: 10.1177/0149206310385943

[ref49] Smith W. K. Lewis M. W. (2011). Toward a theory of paradox: a dynamic equilibrium model of organizing. Acad. Manag. Rev. 36, 381–403. doi: 10.5465/amr.2009.0223

[ref50] Spataro S. E. (2005). Diversity in context: how organizational culture shapes reactions to workers with disabilities and others who are demographically different. Behav. Sci. Law 23, 21–38. doi: 10.1002/bsl.623, PMID: 15706613

[ref51] Spjelkavik Ø. (2012). Supported employment in Norway and in the other nordic countries. J. Vocat. Rehabil. 37, 163–172. doi: 10.3233/JVR-2012-0611

[ref52] Sveinsdottir V. Bull H. C. Evensen S. Reme S. E. Knutzen T. Lystad J. U. (2019). A short history of individual placement and support in Norway. Psychiatr. Rehabil. J. 43, 9–17. doi: 10.1037/prj0000366, PMID: 30945917

[ref53] The Norwegian Government (2014). Agreement in principle for a more inclusive working life (the IA agreement) Oslo: The Ministry of Labor and Social Affairs. Available at: https://www.regjeringen.no/globalassets/upload/asd/dokumenter/2014/ia_20142018/signert_ia_avtale.pdf (Accessed January 4, 2021).

[ref54] United Nations (2020). Take action for the sustainable development goals: United Nations. Available at: https://www.un.org/sustainabledevelopment/sustainable-development-goals/ (Accessed March 10, 2021).

[ref55] van der Noordt M. IJzelenberg H. Droomers M. Proper K. I. (2014). Health effects of employment: A systematic review of prospective studies. Occup. Environ. Med. 71, 730–736. doi: 10.1136/oemed-2013-101891 24556535

[ref56] Waddell G. Burton A. K. (2006). Is Work Good for Your Health and Well-Being? London: TSO.

[ref57] WMA (2013). Declaration of Helsinki - ethical principles for medical research involving human subjects. J. Am. Coll. Dent. 81, 14–18. 25951678

